# Evaluation of a new NS1 rapid diagnostic test using a single acute‐phase serum panel collected during the largest dengue outbreak in Taiwan history in 2015

**DOI:** 10.1002/kjm2.12490

**Published:** 2021-12-21

**Authors:** Li‐Teh Liu, Chun‐Hong Chen, Ping‐Chang Lin, Ching‐Yi Tsai, Miao‐Chen Hsu, Bo‐Yi Huang, Yan‐Yi Tsai, Jih‐Jin Tsai

**Affiliations:** ^1^ Department of Medical Laboratory Science and Biotechnology College of Medical Technology, Chung‐Hwa University of Medical Technology Tainan City Taiwan; ^2^ National Mosquito‐Borne Diseases Control Research Center National Health Research Institutes Zhunan Taiwan; ^3^ National Institute of Infectious Diseases and Vaccinology National Health Research Institutes Zhunan Taiwan; ^4^ Tropical Medicine Center Kaohsiung Medical University Hospital Kaohsiung Taiwan; ^5^ Division of Infectious Diseases, Department of Internal Medicine Kaohsiung Medical University Hospital Kaohsiung Taiwan; ^6^ School of Medicine, College of Medicine Kaohsiung Medical University Kaohsiung Taiwan

**Keywords:** dengue fever, dengue virus, DENV‐2, NS1, rapid diagnosis test

## Abstract

Dengue virus (DENV) infection results mostly from the bites of virus‐carrying Aedes mosquitoes, which results in dengue fever (DF) with or without warning signs, severe dengue, or asymptomatic infections in humans. For point‐of care identification of DENV‐infected patients, a rapid diagnostic test (RDT) for DENV nonstructural protein 1 (NS1) has been developed to achieve early diagnosis and timely clinical management. We evaluated the performance of a new commercially available dengue NS1 RDT AsiaGen Dengue NS1 Antigen Rapid Diagnosis Test using real‐time qRT‐PCR as a reference method and compared the results with SD BIOLINE Dengue NS1 Ag using a single acute‐phase serum panel collected during the largest dengue outbreak in the history of Taiwan in 2015. The results suggested that the sensitivity and specificity of AsiaGen Dengue NS1 Antigen RDT (96.9% and 100%) were similar to those of SD BIOLINE Dengue NS1 RDT (100% and 100%) for detection in the acute phase of DENV‐2 infection. The results suggested that the sensitivity of both RDTs was similar (95.4% ~ 100%) for the sera collected at less than or equal to three days postsymptom onset (PSO). Our results suggested that the two DENV NS1 RDTs used in this study were promising for the timely diagnosis of DENV infection during dengue outbreaks, at least for DENV‐2 in areas where authorized medical laboratories are not available or medical resources are limited. However, the performance of AsiaGen DENV NS1 RDTs in the detection of primary/secondary infections and infection by serotypes of DENV other than DENV‐2 requires further investigation.

## 1 INTRODUCTION

Dengue is thought to be the most important arthropod‐borne disease currently affecting human beings and is predominantly distributed in subtropical and tropical areas worldwide. Dengue is caused by dengue virus (DENV) infection in humans. DENV belongs to the family *Flaviviridae* and genus *Flavivirus* and is classified into DENV‐1 ~ DENV‐4 according to serological tests. The genome of DENV is an ~11 kilobase positive‐sense RNA that encodes three structural proteins and seven nonstructural proteins.^1^ The transmission of DENV to humans occurs through the bites of infected *Aedes aegypti* or *A. albopictus* mosquitoes.^2^ The results of DENV infection include dengue fever (DF) with or without warning signs, severe dengue^3^ or asymptomatic infection, DF, dengue hemorrhagic fever (DHF), and life‐threatening dengue shock syndrome (DSS).^4^ It is estimated that 50–390 million DENV infections occur annually, and the distribution of DENV infection has greatly increased over the past 50 years due to international travel, urbanization, population increases, and global warming.^2^, ^3^ Currently, no authorized antiviral or vaccine is available to treat or prevent DENV infections.

Several diagnostic techniques can be used to diagnose acute DENV infection, such as real‐time quantitative reverse transcription‐polymerase chain reaction (qRT–PCR), anti‐DENV IgM test (Mac‐ELISA) and virus isolation.^5^ These methods are laborious and time‐consuming and may not be suitable for areas with limited medical resources. The development of a nonstructural protein 1 (NS1) rapid diagnosis test (RDT) provides a tool for the rapid diagnosis of DENV infection.^6^, ^7^, ^8^, ^9^, ^10^, ^11^, ^12^, ^13^, ^14^, ^15^ In this study, the performance of a new commercially available dengue NS1 RDT AsiaGen Dengue NS1 Antigen RDT was evaluated using qRT–PCR as a reference method, and the results were compared with SD BIOLINE Dengue NS1 Ag using a single acute‐phase serum panel collected during the largest dengue outbreak in Taiwan's history in 2015.

## 2 MATERIALS AND METHODS

### 2.1 Clinical specimens and ethics

A total of 122 serum samples were used in this study, including 97 samples from confirmed DF patients and 25 healthy donors collected at Kaohsiung Medical University Hospital (KMUH). All serum samples were collected in serum separation tubes (Becton Dickinson, Franklin Lakes, NJ, USA), stored in 200‐μl aliquots at −80°C and subject to DENV qRT–PCR to confirm DENV infection. DENV‐positive samples were investigated by serotype‐specific RT‐PCR to determine the serotype of DENV. All analyses were conducted during the period of 2015–2016. The protocol was approved by the Institutional Review Board of KMUH (KMUHIRB‐FI‐20160076 and KMUHIRB‐960195). Trial registration: Follow‐up of the health condition and investigation of the immunoregulation among patients with or after DF infection, KMUHIRB‐960195, Registered 18 September 2018, and KMUHIRB‐F(I)‐20160076, Registered 12 August 2016, https://kmuh.cims.tw/wiPtms/index.html.

### 2.2 Real‐time qRT‐PCR and serotype‐specific RT‐PCR of DENV

RNA of DENV was extracted from stored serum aliquots and subjected to one‐step SYBR green‐based qRT‐PCR immediately according to the methods obtained from previous reports to detect the presence of DENV in the serum.^16^, ^17^ The standards employed for the positive control were a threshold cycle value (Ct) ≤30 and Tm ≥79°C, and those for the negative control were Ct value ≥40 and Tm <79°C. A Ct value ≤30 or a Tm ≥79°C was considered to indicate sample positivity.^17^, ^18^ The sequences of primers used in the detection and serotyping of DENV were described elsewhere.^16^, ^18^

### 2.3 Sequence analysis

After the serotype of DENV was determined using serotype‐specific RT‐PCR, the sequences of five DENV‐1, twenty randomly selected DENV‐2, and three DENV‐3 conventional serotype‐specific RT‐PCR products were confirmed using the BigDye™ Terminator v3.1 Cycle Sequencing Kit in a 3730xl DNA Analyzer (Applied Biosystems, Foster City, CA, USA).

### 2.4 DENV NS1 antigen detection

The presence of DENV NS1 antigen was analyzed in all sera using an AsiaGen Dengue NS1 Antigen Rapid Test kit (AsiaGen Corp., Taiwan) and SD BIOLINE Dengue NS1 Ag (STANDARD DIAGNOSTICS, INC., Korea) following the manufacturer's instructions. In brief, a 100‐μL mixture of serum and diluent (1:1) was added to the sample pad of the AsiaGen Dengue NS1 Antigen Rapid Test kit. The results were read after 20–30 min. Similarly, 100 μL of a mixture of serum was added to the sample pad of SD BIOLINE Dengue NS1 Ag. The results were read after 15–20 min.

### 2.5 Data analysis

The data were entered in MS Excel 2019 and analyzed using SPSS version 16.0 for Windows (SPSS, Chicago, IL, USA). Sensitivity, specificity, positive predictive value (PPV), negative predictive value (NPV), and Cohen's kappa coefficients (κ) were calculated. A *p* value <0.05 was considered significant.

## 3 RESULTS

### 3.1 Characteristics of participants and serotype‐specific RT‐PCR results

The characteristics of the participants enrolled are illustrated in Table 1. Ninety‐seven cases were confirmed as having an acute infection according to the positive DENV qRT‐PCR results. The serotype in all qRT‐PCR‐positive samples was determined using serotype‐specific primers amplifying the core protein gene. The conventional serotype‐specific RT‐PCR products were subjected to sequencing analysis and were confirmed to be corresponding serotype‐specific DENV sequences to avoid nonspecific products or artifacts in RT‐PCR. The serotype distributions of the 97 qRT‐PCR‐positive samples were DENV1 (5; 5.1%), DENV2 (89; 91.8%), and DENV3 (3; 3.1%). The nondengue cases included 25 samples from healthy donors that were negative for dengue qRT‐PCR.

**TABLE 1:** Characteristics of the participants enrolled and specimens collected in this study

RDT	AsiaGen NS1 and SD NS1	
	Confirmed dengue^b^	Healthy
Variable^a^	(*N* = 97)	(*N* = 25)
Day of illness	2(0–10)	‐
Serotype		
DENV‐1	5 (5.1)	‐
DENV‐2	89 (91.8)	‐
DENV‐3	3 (3.1)	‐
DENV‐4	0 (0)	‐

^a^ Presented as median (range) for day of illness and number (%) for DENV serotype.

^b^ Confirmed dengue was based on positive DENV qRT–PCR results.

### 3.2 Clinical performances of the two DENV NS1 RDTs for the detection of acute dengue infection

The clinical performance of the AsiaGen Dengue NS1 Antigen Rapid Test kit was evaluated and compared with SD BIOLINE Dengue NS1 Ag based on reference qRT‐PCR (Table 2). Based on qRT‐PCR results, SD NS1 RDT was more sensitive (100%) than AsiaGen NS1 RDT for NS1 antigen detection (96.9%). The specificity of the two RDT kits for the NS1 antigen was 100%. Perfect agreement was noted between the two NS1 RDTs and RT‐PCR. However, false‐negative results were observed using AsiaGen NS1 RDT in this study.

**TABLE 2:** Comparison of the performances of two dengue virus (DENV) NS1 RDTs using reference quantitative reverse‐transcriptase polymerase chain reaction (qRT–PCR) as the gold standard

RDT	Total patients (*N*)	Acute dengue cases ^f^ (*N*)	Number positive (*N*)	Sensitivity^a^	Specificity^b^	Kappa value^c^	PPV^d^	NPV^e^
AsiaGen NS1	122	97	94	96.90%	100%	0.9278 (0.8473–1)	100%	89.30%
				(94/97)	(25/25)		(94/94)	(25/28)
SD NS1	122	97	97	100%	100%	1	100%	100%
				(97/97)	(25/25)		(97/97)	(25/25)

^a^ Sensitivity denotes % (number of NS1 positive/number of qRT–PCR positive).

^b^ Specificity is based on 25 samples from negative qRT‐PCR healthy donors.

^c^ Values in parenthesis represent 95% confidence interval.

^d^ PPV: Positive predictive value.

^e^ NPV: Negative predictive value.

^f^ Based on positive DENV qRT‐PCR.

### 3.3 Clinical performances of the two RDT kits using samples obtained the day after the onset of fever

In this study, 97 acute‐phase sera were collected between 2 and 10 days after onset. We divided these sera into two groups. In one group, serum samples were collected at most three days postsymptom onset (PSO). In the other group, serum samples were collected after three days of PSO. The sensitivity of SD NS1 RDTs was 100% in both groups (Table 3). In the case of AsiaGen NS1 RDT, the sensitivity was lower for the sera collected within three days of illness compared with that collected after three days of symptom onset. The results also suggested that the sensitivity of SD NS1 RDT (100%) was greater than that of AsiaGen NS1 RDT (95.4%) using sera collected in less than or equal to 3 days PSO. However, the differences were not statistically significant. Our results agreed with the results by Tricou et al.^15^ given that the sensitivity of NS1 RDT was statistically similar between the two groups. In contrast, several studies concluded that the sensitivity of NS1 RDT was greater for samples collected within three days of PSO.^10^, ^19^, ^20^, ^21^

**TABLE 3:** Sensitivity of the dengue virus (DENV) NS1 rapid tests in sera collected within three days PSO versus those collected later

RDT	AsiaGen NS1	SD NS1
Status^a^	Sensitivity^b^	
Collected ≦ 3 days PSO	95.4% (62/65)	100% (65/65)
Collected >3 days PSO	100% (32/32)	100% (32/32)
*p* value^c^	0.549	‐

^a^ PSO: postsymptom onset.

^b^ Sensitivity denotes % (number of NS1 positive/number of qRT–PCR positive).

^c^ Fisher's exact test.

### 3.4 Sensitivity of the DENV NS1 rapid test for different DENV serotypes

The results in Table 4 show that the sensitivity of SD NS1 RDT in the detection of DENV‐1 to DENV‐3 was 100%. In the case of AsiaGen RDT, the sensitivity was 100% for both DENV‐1 and DENV‐3, whereas the sensitivity was 96.6% for DENV‐2 (*P* > 0.05). However, the DENV‐1 and DENV‐3 sample numbers were too small for statistical analysis.

**TABLE 4:** Sensitivity of rapid diagnostic tests (RDTs) for different dengue virus (DENV) serotypes

	AsiaGen NS1	SD NS1
DENV‐1	100% (5/5)	100% (5/5)
DENV‐2	96.6% (86/89)	100% (89/89)
DENV‐3	100% (3/3)	100% (3/3)
DENV‐4	‐	‐

## 4 DISCUSSION

DENV NS1 plays an important role in the DENV genome replication process, and the pathological mechanism(s) induced by NS1 and its antibodies in dengue diseases have been investigated recently. NS1 overexpression resulted in the upregulation of macrophage migration inhibitory factor (MIF), leading to the upregulation of heparanase 1 (HPA‐1) and endothelial glycocalyx degradation as well as autophagy of endothelial cells. These effects promote hyperpermeability of the endothelium.^22^, ^23^, ^24^ NS1 activates the p38/MAPK^25^ and Toll‐like receptor 4^26^ pathways as well as endocytosis of the NS1 protein in the process of vascular hyperpermeability.^27^ Antibodies against the NS1 antigen interact with the endothelium, resulting in apoptosis mediated by NO production through NF‐κB and ceramide‐regulated glycogen synthase kinase 3β activation.^28^, ^29^ In addition, antibodies against the NS1 antigen enhance the phagocytosis of platelets by macrophages, leading to thrombocytopenia in severe dengue.^30^ The NS1 protein is secreted into the serum during the acute phase of DENV infection and is detectable through days 1–9 PSO. In addition, DENV RNA is not detected by RT‐PCR, making it a suitable viral marker for the diagnosis of acute DENV infection.^7^

RT‐PCR could potentially be used for the diagnosis of dengue for early intervention through clinical care options, outbreak investigations, and disease surveillance. Reverse transcription insulated isothermal PCR (RT‐iiPCR) was performed using pan‐ or serotyping reagents in the mobile system and was able to detect the DENV genome in serum, achieving a sensitivity of 1 to 10 plaque‐forming units per milliliter in our previous research.^31^, ^32^, ^33^ However, the obvious disadvantage of virus genome detection is that the test must be conducted by well‐trained staff.

In this study, we evaluated the clinical performance of two commercial RDTs for DENV NS1 antigen using sera collected from confirmed DF patients and healthy donors during the 2015 dengue outbreak in Kaohsiung City. The statistical results suggested that the sensitivities and specificities of AsiaGen and SD NS1 rapid diagnostic tests were similar (96.9%–100%) using qRT‐PCR as a reference method to detect acute DENV infection. The agreement between the two NS1 RDTs and qRT‐PCR was perfect (kappa value 0.9278 and 1) (Table 2). Two NS1 RDTs exhibited similar sensitivities (95.4%–100%) for the sera samples collected either within three days of illness or three days PSO and for the detection of different serotypes of DENV (96.6%–100%) (Table 3). However, we did not measure the presence of anti‐DENV IgM and IgG antibodies in the sera. The sensitivities and specificities of the two commercial NS1 RDTs for the detection of primary or secondary DENV infection were not available. In addition, 91.8% (*n* = 89) of the sera samples were collected from donors infected with DENV‐2. There were three negative AsiaGen NS1 RDT results in 89 DENV‐2 serum samples. The detection limits of AsiaGen NS1 RDT for DENV‐1 to DENV‐4 were 10, 100, 10, and 10 pg/μL, respectively (information listed in pack insert). This finding might explain why these false results occurred. The false‐negative results of DENV RDT might lead to DENV infections that are not treated. Thus, we suggest that serum should be retested using another DENV NS1 RDT or qRT–PCR if resources are available once the patient is highly suspected to be a dengue patient. Due to the limited number of DENV‐1 and DENV‐3 samples in this study, the sensitivities for the detection of DENV1, DENV‐3, and DENV‐4 need to be further investigated (Table 4).

In conclusion, our results suggested that the sensitivity and specificity of newly available DENV NS1 rapid diagnostic tests from AsiaGen Corp. were similar to the NS1 RDTs from STANDARD DIAGNOSTICS Inc. in the detection of acute DENV infection. Our results suggested that the DENV NS1 RDTs used in this study, which reduce the possibility of human error compared to the cumbersome qRT‐PCR technique, represent promising methods for the timely diagnosis of DENV infection during dengue outbreaks, at least for DENV‐2 in areas where authorized medical laboratories are not available or medical resources are limited. However, the performance of AsiaGen DENV NS1 RDTs in the detection of primary/secondary infections and infection by DENV serotypes other than DENV‐2 needs to be investigated further.

## CONFLICT OF INTEREST

The authors declare that the research was conducted in the absence of any commercial or financial relationships that could be construed as a potential conflict of interest.
